# Lightweight, Flexible Cellulose-Derived Carbon Aerogel@Reduced Graphene Oxide/PDMS Composites with Outstanding EMI Shielding Performances and Excellent Thermal Conductivities

**DOI:** 10.1007/s40820-021-00624-4

**Published:** 2021-03-16

**Authors:** Ping Song, Bei Liu, Chaobo Liang, Kunpeng Ruan, Hua Qiu, Zhonglei Ma, Yongqiang Guo, Junwei Gu

**Affiliations:** 1 MOE Key Laboratory of Material Physics and Chemistry under Extraordinary Conditions, Shaanxi Key Laboratory of Macromolecular Science and Technology, School of Chemistry and Chemical Engineering, Northwestern Polytechnical University, Xi’an, 710072 P. R. China; 2 Xi’an ESWIN Silicon Wafer Technology Co. Ltd, Xi’an, 710100 P. R. China

**Keywords:** Polydimethylsiloxane, Electromagnetic interference shielding, Cellulose carbon aerogel, Reduced graphene oxide

## Abstract

In order to ensure the operational reliability and information security of sophisticated electronic components and to protect human health, efficient electromagnetic interference (EMI) shielding materials are required to attenuate electromagnetic wave energy. In this work, the cellulose solution is obtained by dissolving cotton through hydrogen bond driving self-assembly using sodium hydroxide (NaOH)/urea solution, and cellulose aerogels (CA) are prepared by gelation and freeze-drying. Then, the cellulose carbon aerogel@reduced graphene oxide aerogels (CCA@rGO) are prepared by vacuum impregnation, freeze-drying followed by thermal annealing, and finally, the CCA@rGO/polydimethylsiloxane (PDMS) EMI shielding composites are prepared by backfilling with PDMS. Owing to skin-core structure of CCA@rGO, the complete three-dimensional (3D) double-layer conductive network can be successfully constructed. When the loading of CCA@rGO is 3.05 wt%, CCA@rGO/PDMS EMI shielding composites have an excellent EMI shielding effectiveness (EMI SE) of 51 dB, which is 3.9 times higher than that of the co-blended CCA/rGO/PDMS EMI shielding composites (13 dB) with the same loading of fillers. At this time, the CCA@rGO/PDMS EMI shielding composites have excellent thermal stability (*T*_*HRI*_ of 178.3 °C) and good thermal conductivity coefficient (*λ* of 0.65 W m^-1^ K^-1^). Excellent comprehensive performance makes CCA@rGO/PDMS EMI shielding composites great prospect for applications in lightweight, flexible EMI shielding composites.

**Graphic abstract:**

**Supplementary Information:**

The online version contains supplementary material available at 10.1007/s40820-021-00624-4.

## Highlights

- Cellulose aerogels were prepared by hydrogen bonding driven self-assembly, gelation and freeze-drying.
- The skin-core structure of CCA@rGO aerogels can form a perfect three-dimensional bilayer conductive network.
- Outstanding EMI SE (51 dB) is achieved with 3.05 wt% CCA@rGO, which is 3.9 times higher than that of the co-blended composites.

**Supplementary Information:**

The online version contains supplementary material available at 10.1007/s40820-021-00624-4.

## Graphical abstract

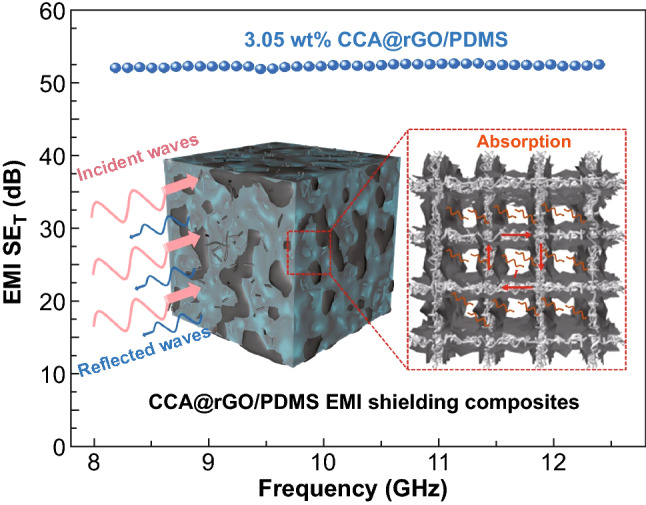

## Introduction

While electronic and electrical equipment have brought great convenience to our lives, they have also caused increasingly serious electromagnetic pollution, such as electronic noise, electromagnetic interference and radio frequency interference [1–3]. Electromagnetic waves not only couple and interfere with the normal use of other electronic components, making it impossible for electronic equipment to function properly and posing serious threat to information security, but also affect human health. Studies have shown that when people are exposed to electromagnetic radiation for a long time, the risk of diseases such as cancer, heart disease, skin problems, headaches and other mild or acute diseases will increase. Therefore, the design and development of lightweight, economical and efficient EMI shielding materials are imperative to address the problems of electromagnetic pollution [4–6].

Compared with traditional metal-based EMI shielding composites, polymer-based EMI shielding composites have attracted much attention from the scientific and industrial communities due to their lightweight, high specific strength, easy molding and processing, excellent chemical stability, low cost and good sealing properties [7–9] Commonly used polymer matrixes are epoxy resin, phenolic resin, polyvinylidene fluoride (PVDF) and polydimethylsiloxane (PDMS). Among them, PDMS has good mechanical properties, high and low temperature resistance, excellent weather resistance, chemical stability and easy processing and molding characteristics, widely used in many fields such as aerospace, automotive industries and microelectronics [10–12]. In addition, PDMS has excellent flexibility compared to rigid matrixes such as epoxy resins and can meet the requirements of wearable electronic devices for flexibility in materials. In recent years, PDMS-based EMI shielding composites have made certain research progress, but to achieve the desired EMI shielding effectiveness (EMI SE) usually requires high filler loading, which seriously affects the cost, processability and mechanical properties, largely limiting the application of PDMS-based EMI shielding composites in the field of microelectronics, aircraft and spacecraft [13–15]. Therefore, the development of PDMS-based EMI shielding composites with excellent EMI shielding performances at low filler loading is a research hotspot.

As the abundant renewable bioenergy on the earth, biomass (such as straw, wood, sugarcane and cotton) is easy and fast to prepare from a wide variety of sources [16–19]. The preparation of biomass-based carbon aerogel/polymer composites by certain methods has a wide range of applications in the fields of flexible conductive materials, supercapacitors, energy storage materials and EMI shielding materials [20–22]. Shen et al. [23] prepared aerogel (Cs)/epoxy EMI shielding composites by carbonizing natural wood at 1200 °C to obtain Cs and then backfilling with epoxy resin. The results showed that the electrical conductivity (*σ)* and EMI SE_T_ of the Cs/epoxy EMI shielding composites reached 12.5 S m^−1^ and 28 dB, respectively. Li et al. [24] prepared aerogel-like carbon (ALC)/PDMS EMI shielding composites by hydrothermal carbonization of sugarcane to obtain ALC, followed by backfilling with PDMS. The results showed that the EMI SE_T_ of ALC/PDMS EMI shielding composites reached 51 dB with the thickness of 10 mm. Ma et al. [25] obtained straw-derived carbon (SC) aerogel by carbonizing wheat straw at 1500 °C and then prepared SC/epoxy EMI shielding composites by backfilling with epoxy resin. The results showed that the EMI SE_T_ of SC/epoxy EMI shielding composites reached 58 dB with the thickness of 3.3 mm.

It has been shown that the EMI SE of biomass-based carbon aerogel/polymer EMI shielding composites can be further enhanced by compounding the carbon aerogel with highly conductive materials (such as silver wire, MXene and graphene) or magnetic materials (such as iron, cobalt, nickel and their oxides) [26, 27]. The introduction of reduced graphene oxide (rGO) into cellulose carbon aerogels (CCA) can further improve the 3D conductive network and significantly enhance the *σ* of biomass-based carbon aerogel/polymer EMI shielding composites, thus effectively improving their EMI SE [28]. Zeng et al. [29] prepared ultra-lightweight and highly elastic rGO/lignin-derived carbon (LDC) aerogel EMI shielding composites by freeze-drying. The results showed that the EMI SE_T_ of the rGO/LDC aerogel EMI shielding composites reached 49 dB with the thickness of 2 mm. Wan et al. [30] prepared ultra-lightweight cellulose fiber (CF)/rGO aerogel EMI shielding composites by freeze-drying and carbonization. The results showed that the EMI SE_T_ of the CF/rGO aerogel EMI shielding composite reached 48 dB with the thickness of 5 mm. In our previous research, Gu et al. [31] prepared annealed sugarcane (ACS) by hydrothermal method and annealing, followed by vacuum-assisted impregnation to prepare ASC/rGO aerogel EMI shielding composites. The results showed that the EMI SE_T_ of ASC/rGO aerogel EMI shielding composites reached 53 dB with the thickness of 3 mm.

In this paper, NaOH/urea solution was used to dissolve cotton *via* hydrogen bond driving self-assembly to obtain cellulose solution and then CA was prepared by combining gelatinization, freeze-drying. The optimized CA was impregnated into GO solution, freeze-dried to produce CA@GO aerogel with GO loaded on CA backbone, then carbonized at high temperature to produce CCA@rGO aerogel with rGO loaded on CCA backbone and finally backfilled with PDMS to produce CCA@rGO/PDMS EMI shielding composites. On this basis, the effects of CCA and rGO loading on the electrical conductivities, EMI SE, thermal conductivities, mechanical and thermal properties of CCA@rGO/PDMS EMI shielding composites were investigated.

## Experimental

### Preparation of CA

NaOH/urea solution was used to dissolve the cotton by hydrogen bond driving self-assembly to obtain the cellulose solution. The process was described below. The NaOH/urea solution (NaOH/urea/water = 7/12/81, wt/wt/wt) was first prepared and pre-cooled to 0 °C. An appropriate amount of dried cotton was then weighed and immersed in the pre-cooled solution (cotton/pre-cooled solution = 1/99, 2/98, 3/97, 4/96 and 5/95, wt/wt) and mechanically stirred in an ice bath (0 °C) for 48 h to obtain the cellulose solution, and the corresponding solution concentrations were 1 wt%, 2 wt%, 3 wt%, 4 wt% and 5 wt%, respectively. The cellulose hydrogel was obtained by adding a certain amount of cellulose solution to a three-necked flask equipped with a condensing unit and heating to 70 °C for 24 h. The cellulose hydrogel was soaked in deionized water, changed at 12 h intervals to pH=7 and frozen in liquid nitrogen (−56 °C) and freeze-dried for 72 h to obtain cellulose aerogel (CA).

### Preparation of CCA@rGO/PDMS

**Fig. 1:**
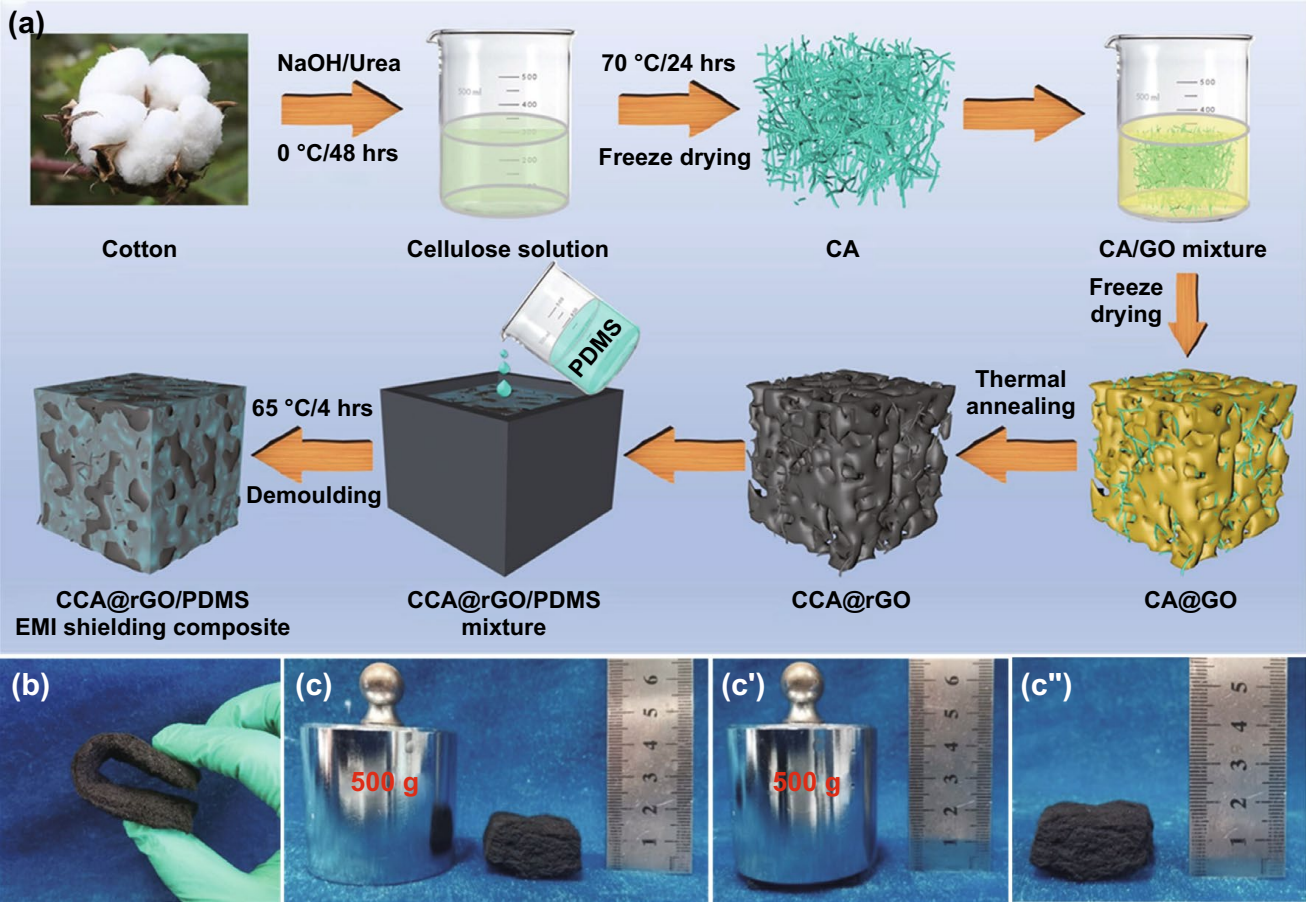
Schematic illustration of the fabrication procedure for CCA@rGO/PDMS EMI shielding composites (**a**), illustration of the flexibility (**b**) and resilience (**c-c’’**) of CCA@rGO aerogel

GO was prepared by modified Hummers method, and a range of GO solutions at different concentrations (2.5, 5, 7.5 and 10 mg mL^−1^) were configured. The CA@GO was prepared by impregnating the pre-prepared CA into the above aqueous GO solution, evacuating until no air bubbles emerged, then freezing (−56 °C) and freeze-drying for 72 h to obtain CA@GO with GO loaded on the CA backbone. After carbonization at 1500 °C for 2 h under nitrogen atmosphere, CCA@rGO with rGO supported on the CCA framework was obtained. The prepared CCA@rGO foam has excellent flexibility and can withstand bending deformations up to 180 °C (Fig. 1b). It also has excellent mechanical load-bearing performance and resilience. It can load 500 g weights, and the original shape can be restored immediately after the weights are removed (Fig. 1c-c'').

Weigh a certain amount of PDMS and n-hexane (PDMS/n-hexane=1/2, vol/vol), and mechanically stir at room temperature for 30 min to obtain the PDMS/n-hexane solution. The CCA@rGO was placed in the mould and the portion of the PDMS/n-hexane solution was first poured into the mould and vacuum impregnated at room temperature until there were no bubbles. The PDMS/n-hexane solution was then poured into the mould and the vacuum impregnation was continued at room temperature until there were no bubbles. This was repeated until the PDMS/n-hexane solution had completely submerged the CCA@rGO and the temperature was raised to 65 °C for 4 h. The CCA@rGO/PDMS EMI shielding composites were obtained by simple processing after natural cooling to room temperature. The schematic diagram is shown in Fig. 1a.

At the same time, CCA@rGO was crushed to obtain P(CCA@rGO). A series of P(CCA@rGO)/PDMS EMI shielding composites with the same amount of CCA@rGO as the CCA@rGO/PDMS EMI shielding composites were prepared by controlling the amount of P(CCA@rGO) added. The prepared CA was carbonized at 1500 °C under nitrogen atmosphere for 2 h to obtain cellulose carbon aerogel (CCA). The same PDMS casting process was adopted to obtain CCA/PDMS EMI shielding composites

## Results and Discussion

### Characterization on CA, CCA, CA@GO and CCA@rGO

Figure S1a illustrates the thermogravimetric analyses curves of CA and CCA. It can be seen that CA has a significant thermal weight loss process from 200 to 400 °C, and the residual carbon rate at 1000 °C is 5.4%, which is mainly attributed to the low thermal stability of CA due to the rich hydrogen and oxygen elements in the cellulose molecular chains inside CA. In contrast, CCA has no significant thermal weight loss, with a residual carbon percentage of 98.6% at 1000 °C. This is mainly attributed to the fact that after carbonization at 1500 °C, CCA removes most of the oxygen-containing functional groups and has a very high degree of carbonization. Figure S1b shows the Fourier transform infrared spectroscopy (FTIR) spectra of CA and CCA. It can be seen that in the FTIR spectra of CA, 3358, 2903, 1470~1320, 1450, 1173 and 1058 cm^−1^ are the vibrational peaks of O–H, C–H, C–H, C=O, C–O–H and C–O–C, respectively. In contrast, in the FTIR spectra of CCA, the characteristic absorption peaks of the above functional groups almost all disappear, mainly attributed to the chemical inertness of CCA, which makes it show almost no characteristic absorption peaks. Figure S1c shows the X-ray diffraction (XRD) spectra of CA and CCA. We can see that the main diffraction peaks of CA appear at 14.7° (101), 16.7° (101) and 22.5° (002), which are characteristic diffraction peaks of type I cellulose [32]. The main diffraction peaks of CCA appear at 23.5° (002) and 43.8° (100), which are formed by the specific reflection of graphitic carbon on the (002) and (100) planes and are mainly attributed to the high-temperature carbonization of CA into CCA containing graphitic carbon. Figure S1d shows the Raman spectra of CA and CCA. The D peak (1340 cm^−1^), G peak (1590 cm^-1^) and 2D peak (2500~3000 cm^−1^) correspond to the defective/disordered carbon, the tangential planar stretching vibration peak of *sp*^2^-hybridized carbon and the characteristic peak of graphitic carbon, respectively. The Raman spectrum of CA only has the G peak, which is attributed to the regularity of the cellulose network within CA. The Raman spectrum of CCA contains both D and G peaks, which is attributed to the production of irregular graphitic carbon in CCA during the high-temperature carbonization. In addition, the Raman spectrum of CCA also shows a 2D peak, which further evidence of the production of graphitic carbon. Figure S1e shows the X-ray photoelectron spectroscopy (XPS) spectrum of CA and CCA. Both CA and CCA have obvious C 1s (284.0 eV) and O 1s (530.0 eV) peaks. The C 1s peak of CA is weaker and the O 1s peak is stronger, with a C/O ratio of 1.61. Compared to CA, the C 1s peak of CCA is more intense and the O 1s peak is less intense, with a corresponding increase in C/O ratio to 13.90. This is mainly due to the gradual removal of oxygen-containing functional groups and the carbonization of the cellulose molecular chains under high-temperature conditions. In addition, the three characteristic peaks in the high-resolution C 1s spectra of CA and CCA (Fig. S1e’) are 284.6 eV (sp^2^C-sp^2^C), 285.6 eV (sp^3^C-sp^3^C) and 287 eV (C=O), respectively. Compared to CA, the characteristic peaks of sp^2^C-sp^2^C and sp^3^C-sp^3^C of CCA are enhanced, while the characteristic peak of C=O is weakened, mainly attributed to the high-temperature removal of most of the C=O and conversion to graphitic carbon [33]. Figure S2 further supports the removal of oxygen-containing functional groups on CCA.

**Fig. 2:**
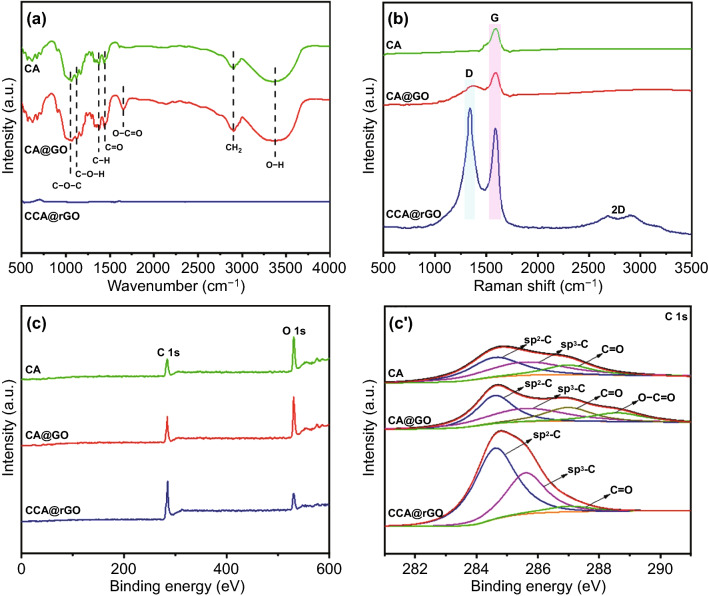
FTIR (**a)**, Raman (**b**), XPS spectra (**c**) and high-resolution C 1s (**c’**) of CA, CA@GO and CCA@rGO

Figure 2a shows the FTIR spectra of CA, CA@GO and CCA@rGO. In the FTIR spectra of CA, 3358 cm^−1^ is the stretching vibration peak of O–H, 2903 cm^−1^ is the stretching vibration peak of C–H in CH_2_, 1470~1320 cm^-1^ is the bending vibration peak of C–H, 1450 cm^-1^ is the stretching vibration peak of C=O, 1173 cm^−1^ is the stretching vibration peak of C–O–H, and 1058 cm^-1^ is the C–O–C stretching vibration peak. In the FTIR spectra of CA@GO, in addition to the characteristic peaks mentioned above, a stretching vibration peak of O–C=O at 1652 cm^-1^ appears, attributed to the introduction of GO [34]. In contrast, in the FTIR spectra of CCA@rGO, these functional groups almost completely disappear, mainly due to the fact that CCA@rGO is chemically inert so that it shows almost no characteristic absorption peaks. Figure 2b shows the Raman spectra of CA, CA@GO and CCA@rGO. Only the G peak is present in the Raman spectrum of CA, which is attributed to the regularity of the cellulose network within CA. A faint D peak starts to appear in the Raman spectrum of CA@GO, which is attributed to the irregular graphitic carbon structure in CA@GO due to the introduction of GO [28]. The Raman spectrum of CCA@rGO contains D peaks, G peaks and 2D peaks, and the D peaks are stronger than the G peaks. This is mainly attributed to the irregular graphitic carbon produced by CCA during the carbonization. At the same time, GO is reduced to rGO by thermal annealing, resulting in a large amount of irregular graphitic carbon structure in CCA@rGO. Figure 2c shows the XPS spectra of CA, CA@GO and CCA@rGO. The C 1s (284.0 eV) and O 1s (530.0 eV) peaks are evident in CA, CA@GO and CCA@rGO. The C 1s peaks are weaker and the O 1s peaks are stronger in CA and CA@GO. Compared to CA and CA@GO, CCA@rGO has a significantly higher C 1s peak intensity and a significantly lower O 1s peak intensity, which is mainly attributed to the gradual removal of oxygen-containing functional groups, the carbonization of cellulose molecular chains and the reduction of GO to rGO under high-temperature conditions [35]. In addition, the high-resolution C 1s spectra of CA and CCA@rGO (Fig. 2c’) have three characteristic peaks: 284.6 eV (sp^2^C-sp^2^C), 285.6 eV (sp^3^C-sp^3^C) and 287 eV (C=O), respectively. In contrast to CA, a new characteristic peak of 288.6 eV appears in the high-resolution C 1s spectrum of CA@GO, which is characteristic of O–C=O in GO. Compared with CA@GO, the characteristic peaks of sp^2^C-sp^2^C and sp^3^C-sp^3^C of CCA@rGO are enhanced, the characteristic peak of O–C=O disappears, and the characteristic peak of C=O is very weak, which is mainly due to the removal of most of the C=O by CCA and conversion to graphitic carbon and the reduction of GO to rGO [36].

### Morphologies of CA, CCA, CCA@rGO and CCA@rGO/PDMS

**Fig. 3:**
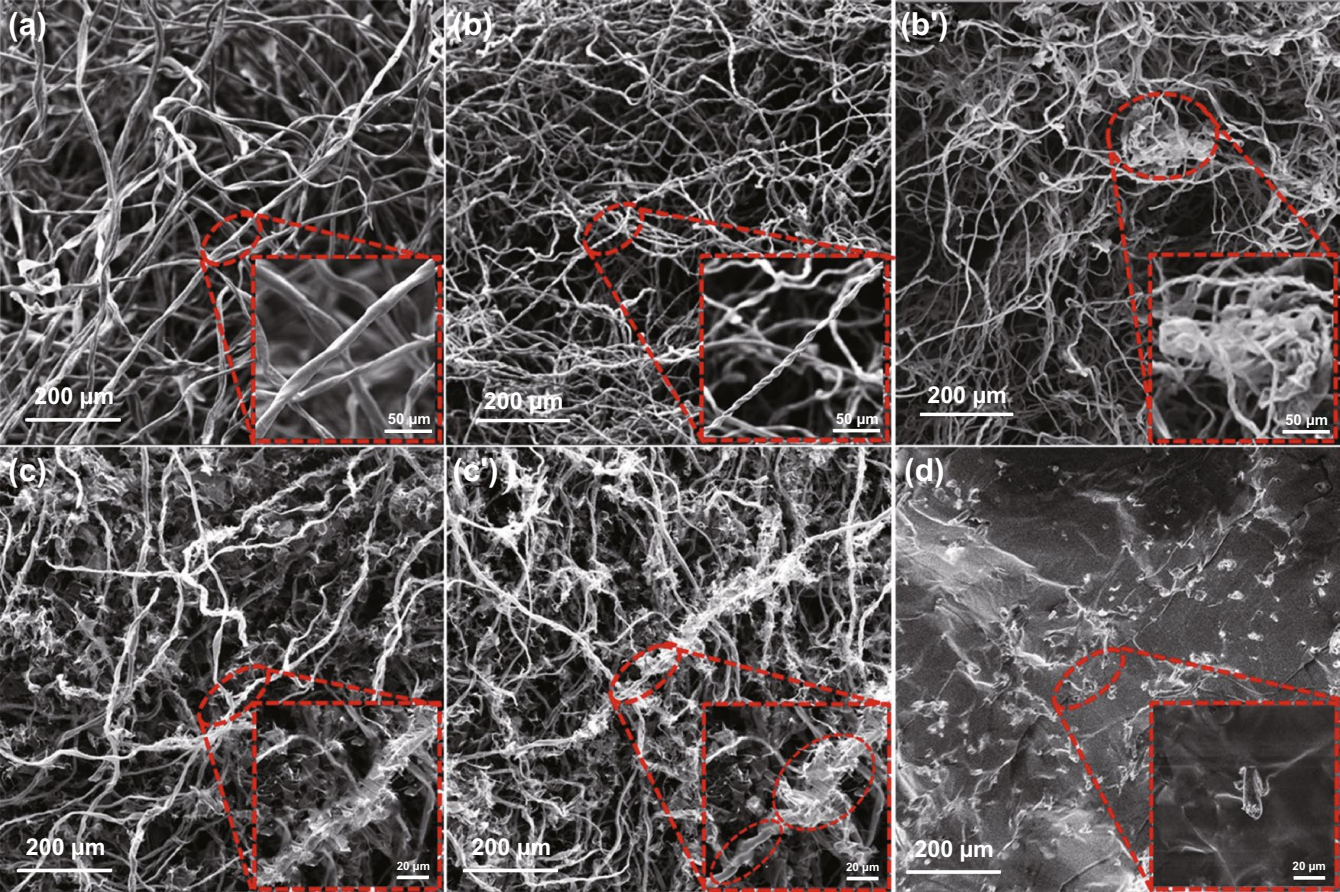
SEM images of CA (**a**), CCA (**b-b’**), CCA@rGO (**c-c’**) and the CCA@rGO/PDMS EMI shielding composites (**d**)

Figure 3 shows SEM images of the CA, CCA, CCA@rGO and CCA@rGO/PDMS EMI shielding composites. Figure 3a shows that the CA is a kind of 3D aerogel made of cellulose fibers entwined with each other, and the individual fibers have the relatively regular circular rodlike structure with the diameter of approximately 12 µm. When the mass ratio of cotton to pre-cooled solution is 4:96, CCA is the 3D carbon aerogel formed by fibers lapping onto each other. But unlike CA, the single fibers of CCA have the twisted twist-like structure and are approximately 6 µm in diameter (Fig. 3b), which is mainly attributed to the removal of oxygen-containing functional groups and the carbonization of the cellulose. When the mass ratio of cotton to pre-cooled solution is 5:95, the tangling of fibers within the CCA is more severe (Fig. 3b’). This is due to the limited solubility of the NaOH/urea solution on the cotton, resulting in the tangling of fibers within the CCA. As shown in Fig. 3c, when the GO solution concentration is 7.5 mg mL^−1^, the CCA@rGO is the homogeneous network structure, with the CCA forming the main framework of the CCA@rGO and the rGO lamellae completely wrapping the fibers, forming the skin-core structure similar to that of a cable. The rGO is similar to the skin and is densely wrapped around the CCA fibers to provide sufficient structural stability for CCA@rGO. The CCA is similar to the core and is wrapped by rGO sheets to provide attachment points and support for the rGO sheets. When the GO solution concentration is 10 mg mL^-1^, rGO is agglomerated in CCA@rGO, CCA is not uniformly wrapped and CCA@rGO has an uneven network structure (Fig. 3c’). This is due to the high viscosity of the GO solution, which limits its diffusion inside the CA and eventually leads to the agglomeration of rGO in the CCA. After backfilling with PDMS, the skin-core structure of CCA@rGO is well preserved, and the 3D double-layer conductive network structure of CCA@rGO is not significantly damaged (Fig. 3d), and PDMS is more uniformly dispersed in the gaps of the 3D conductive network of CCA@rGO. At the same time, PDMS is uniformly dispersed in the gaps of the 3D conducting network of CCA@rGO.

### Electrical Conductivities and EMI Shielding Performances

**Fig. 4:**
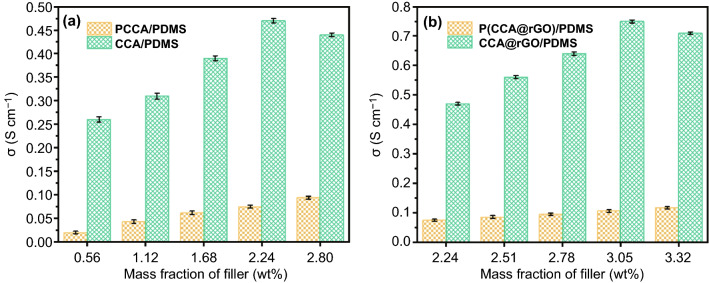
*σ* of the PCCA/PDMS and CCA/PDMS EMI shielding composites (**a**) and P(CCA@rGO)/PDMS and CCA@rGO/PDMS EMI shielding composites (**b**)

Figure 4a shows the *σ* of PCCA/PDMS and CCA/PDMS EMI shielding composites. It can be seen that the *σ* of PCCA/PDMS EMI shielding composites shows a gradual increase with the increase in the amount of PCCA. When the loading of PCCA is 2.80 wt%, the *σ* of the PCCA/PDMS EMI shielding composites reaches 0.094 S cm^-1^. This is mainly due to the fact that the conductive network inside the PCCA/PDMS EMI shielding composites is gradually improved with the increase in PCCA. As the loading of CCA increases, the *σ* of the CCA/PDMS EMI shielding composites tends to increase and then decrease. When the loading of CCA is 2.24 wt%, the CCA/PDMS EMI shielding composites have the largest *σ* value (0.47 S cm^−1^). This is mainly attributed to the gradual improvement of the CCA–CCA conductive network within the CCA/PDMS EMI shielding composites with increasing CCA loading [37]. However, the further increase in the amount of CCA causes the fibers within the CCA to twist into knots, which is detrimental to the formation of the complete conductive pathway and thus has negative impact on the *σ*. In addition, the *σ* of the CCA/PDMS EMI shielding composites is consistently much larger than that of the PCCA/PDMS EMI shielding composites for the same amount of CCA or PCCA [38]. At a CCA loading of 2.24 wt%, *σ* for the CCA/PDMS EMI shielding composites (0.47 S cm^−1^) is 6.3 times greater than that of the PCCA/PDMS EMI shielding composites (0.075 S cm^-1^) with the same loading of PCCA. Mainly due to the random distribution of PCCA in the PCCA/PDMS EMI shielding composites, which is difficult to form the effective PCCA–PCCA conductive network. Within the CCA/PDMS EMI shielding composites, CCA has the more complete 3D conductive network structure, giving it an even better *σ.*

Figure 4b shows the *σ* comparison diagram of P(CCA@rGO)/PDMS and CCA@rGO/PDMS EMI shielding composites. It can be seen that the *σ* of the P(CCA@rGO)/PDMS EMI shielding composites tends to increase as the loading of P(CCA@rGO) increases. The *σ* of the P(CCA@rGO)/PDMS EMI shielding composites reaches 0.117 S cm^-1^ at a P(CCA@rGO) loading of 3.32 wt%, mainly due to the gradual improvement of the conductive network inside the P(CCA@rGO)/PDMS EMI shielding composites with the increasing P(CCA@rGO) loading. With the increase in the loading of CCA@rGO, the *σ* of the CCA@rGO/PDMS EMI shielding composites tends to increase and then decrease [39]. When the loaidng of CCA@rGO is 3.05 wt%, the CCA@rGO/PDMS EMI shielding composites have the largest *σ* value (0.75 S cm^-1^), which is 59.6% higher than the *σ* (0.47 S cm^−1^) of the CCA/PDMS EMI shielding composites (2.24 wt% CCA). This is mainly due to the fact that the rGO wrapped around the CCA gradually forms the second conductive network as the amount of rGO increases based on the first conductive network (2.24 wt% CCA). The synergy of the two conductive networks results in the gradual improvement of the internal conductive network of the CCA@rGO/PDMS EMI shielding composites and the consequent increase in its *σ* [40]. However, as the amount of rGO increases further, the rGO is prone to agglomeration inside the CCA@rGO and the CCA is not uniformly wrapped, resulting in an imperfect second conductive network, which has negative impact on the *σ*. It can also be seen that the *σ* of the CCA@rGO/PDMS EMI shielding composites is consistently much greater than that of the P(CCA@rGO)/PDMS EMI shielding composites for the same loading of CCA@rGO and P(CCA@rGO). When the loading of CCA@rGO is 3.05 wt%, the *σ* of the CCA@rGO/PDMS EMI shielding composites (0.75 S cm^-1^) is 7.1 times higher than that of the P(CCA@rGO)/PDMS EMI shielding composites (0.106 S cm^-1^) with the same loading of P(CCA@rGO). Mainly due to the random distribution of P(CCA@rGO) in the P(CCA@rGO)/PDMS EMI shielding composites, which makes it difficult to form an effective P(CCA@rGO)-P(CCA@rGO) conductive network through point–point laps [41]. In the CCA@rGO/PDMS EMI shielding composites, the CCA@rGO has the more complete 3D conductive network. At the same time, the rGO sheet is wrapped around the CCA fibers to form the double-layer conductive network with the skin-core structure. The CCA–CCA (wire–wire), CCA–rGO (wire–surface) and rGO–rGO (surface–surface) laps form the very complete 3D double-layer conductive network, giving it an even better *σ.*

**Fig. 5:**
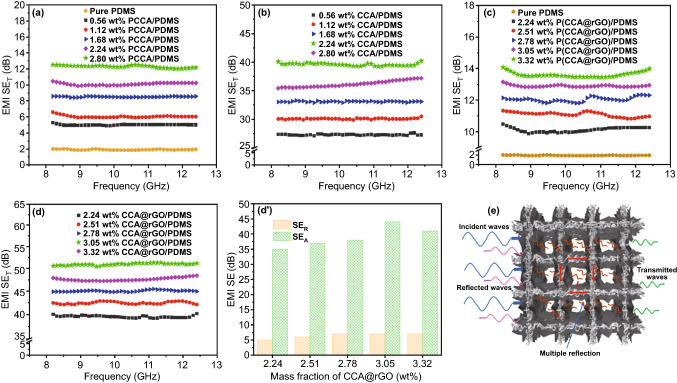
EMI SE_T_ of the PCCA/PDMS EMI shielding composites (**a**), EMI SE_T_ of the CCA/PDMS EMI shielding composites (**b**), EMI SE_T_ of the P(CCA@rGO)/PDMS EMI shielding composites (**c**), EMI SE_T_ (**d**), EMI SE_A_ and SE_R_ (**d’**) of the CCA@rGO/PDMS EMI shielding composites, schematic illustration of EMI shielding mechanism (**e**)

Figure 5 shows the comparison graph of the EMI shielding effectiveness (EMI SE) results of PCCA/PDMS, CCA/PDMS, P(CCA@rGO)/PDMS and CCA@rGO/PDMS EMI shielding composites. As shown in Fig. 5a, the EMI SE_T_ of the PCCA/PDMS EMI shielding composites tends to increase as the loading of PCCA increases. When the loading of PCCA is 2.80 wt%, the EMI SE_T_ of the PCCA/PDMS EMI shielding composites is 12 dB. This is mainly due to the PCCA–PCCA conductive network within the PCCA/PDMS EMI shielding composites gradually improving with increasing PCCA loading, resulting in an increased ability to reflect and absorb incident electromagnetic waves, which is reflected in the increase in EMI SE_T_ value [42]. Figure 5b shows that as the loading of CCA increases, the EMI SE_T_ of the CCA/PDMS EMI shielding composite material first increases and then decreases. When the loading of CCA is 2.24 wt%, the CCA/PDMS EMI shielding composites have the best EMI SE_T_ (40 dB), which is 20 times than that of pure PDMS (2 dB). This is because the density of the CCA–CCA conductive network within the CCA/PDMS EMI shielding composites increases as the amount of CCA increases [43]. At the same time, the two-phase interface with the PDMS matrix increases, resulting in enhanced conductive losses, impedance mismatch and interfacial polarization losses between the incident electromagnetic waves and the CCA–CCA conductive network, thus significantly improving the EMI SE_T_ of the CCA/PDMS EMI shielding composite [44]. However, when the amount of CCA is too high, the fibers in CCA tend to twist into knots, which reduces the conductive network density of CCA and reduces the two-phase interface between CCA and PDMS substrate, thus reducing its EMI SE_T_. As shown in Fig. 5c, the EMI SE_T_ of the P(CCA@rGO)/PDMS EMI shielding composites tends to increase gradually as the loading of P(CCA@rGO) increase. The EMI SE_T_ of P(CCA@rGO)/PDMS EMI shielding composites is 14 dB when the loading of P(CCA@rGO) is 3.32 wt%. This is mainly due to the gradual improvement of the conductive network inside the P(CCA@rGO)/PDMS EMI shielding composites with the increase in the loading of P(CCA@rGO), which leads to the enhancement of its ability to reflect and absorb the incident electromagnetic waves, manifested in the increase in the EMI SE_T_ [45, 46]. Figure 5d shows that the EMI SE_T_ of CCA@rGO/PDMS EMI shielding composites tend to increase and then decrease as the loading of CCA@rGO increase. When the loading of CCA@rGO is 3.05 wt%, the CCA@rGO/PDMS EMI shielding composites have the best EMI SE_T_ (51 dB), which is 27.5% higher than the EMI SE_T_ (40 dB) of the CCA/PDMS EMI shielding composites (2.24 wt% CCA) and 25.5 times higher than that of the pure PDMS (2 dB). This is because as the amount of CCA@rGO increases, the CCA (first conductive network) wrapped with rGO gradually forms the perfect second conductive network, and the skin-core structure of CCA@rGO makes the two conductive networks work together to form the perfect 3D double-layer conductive network [47]. At the same time, the interfaces between rGO and CCA, rGO and rGO, and CCA@rGO and PDMS matrix are increased, so that the conductive loss, impedance mismatch and interface polarization loss between CCA@rGO/PDMS EMI shielding composite and incident electromagnetic waves are enhanced, which significantly improves the EMI SE_T_ of CCA@rGO/PDMS EMI shielding composites. However, when the loading of rGO is too high, rGO tends to agglomerate in CCA@rGO, resulting in an imperfect second conductive network and reducing the conductive network density. At the same time, it reduces the two-phase interface between CCA@rGO and PDMS matrix, which adversely affects the EMI SE_T_ of CCA@rGO/PDMS EMI shielding composites [48].

Combining Fig. 5c, d also shows that the EMI SE_T_ of CCA@rGO/PDMS EMI shielding composites is always better than that of P(CCA@rGO)/PDMS EMI shielding composites at the same CCA@rGO and P(CCA@rGO) loading. When the amount of CCA@rGO is 3.05 wt%, the EMI SE_T_ of CCA@rGO/PDMS EMI shielding composites is 51 dB, which is 3.9 times higher than that of P(CCA@rGO)/PDMS EMI shielding composites (13 dB) with the same loading of filler. This is because the conductive fillers in P(CCA@rGO)/PDMS EMI shielding composites are randomly distributed, and the efficiency of lap bonding through P(CCA@rGO)-P(CCA@rGO) (point–point) is extremely low [49]. At the same time, P(CCA@rGO) has high surface energy and are prone to agglomeration within the PDMS matrix, which makes it difficult to form an effective conductive network, thus affecting the reflectivity and dissipation ability of the P(CCA@rGO)/PDMS EMI shielding composites for incident electromagnetic waves, and therefore, its EMI SE_T_ enhancement effect is poor [50]. For the CCA@rGO/PDMS EMI shielding composites, the skin-core structure allows CCA@rGO to form the 3D double-layer conductive network structure with a high conductive network density, which enhances the conductive loss and impedance mismatch between the incident electromagnetic waves and the CCA@rGO/PDMS EMI shielding composites (Fig. 5e). Meanwhile, the introduction of rGO leads to more two-phase interfaces between rGO and CCA, rGO and rGO, and CCA@rGO and PDMS matrix, which significantly improves the interfacial polarization loss capability of CCA@rGO/PDMS EMI shielding composites to incident electromagnetic waves [51]. The synergistic effect of the two aspects makes the CCA@rGO/PDMS EMI shielding composites have relatively stronger reflection, scattering and absorption of incident electromagnetic waves, so that their EMI SE_T_ is consistently better than that of P(CCA@rGO)/PDMS and CCA@rGO/PDMS EMI shielding composites [52].

Figure 5d’ shows that the EMI SE_A_ and EMI SE_R_ of CCA@rGO/PDMS EMI shielding composites also tend to increase and then decrease as the loading of CCA@rGO increases. When the loading of CCA@rGO is 3.05 wt%, the EMI SE_R_ and EMI SE_A_ of CCA@rGO/PDMS EMI shielding composites reach the maximum values of 7 dB and 44 dB, respectively. This is because the continuous increase in CCA@rGO provides more mobile charge, which enhances the impedance mismatch between the CCA@rGO/PDMS EMI shielding composites and the incident electromagnetic wave, hence the EMI SE_R_ increase [53]. Meanwhile, the CCA@rGO-CCA@rGO double-layer conductive network is gradually improved with the increase in CCA@rGO loading, which can provide more carriers for dissipating electromagnetic waves, so its EMI SE_A_ is improved [54]. However, as the loading of CCA@rGO increases further, rGO tends to agglomerate inside CCA@rGO and CCA is not uniformly wrapped, reducing the internal conductive network density of CCA@rGO/PDMS EMI shielding composites and decreasing the two-phase interface between CCA@rGO and PDMS matrix [55]. This weakens the ability of the CCA@rGO/PDMS EMI shielding composite to reflect, scatter and absorb incident electromagnetic waves, resulting in lower EMI SE_R_ and EMI SE_A_.

### Thermal Conductivities

**Fig. 6:**
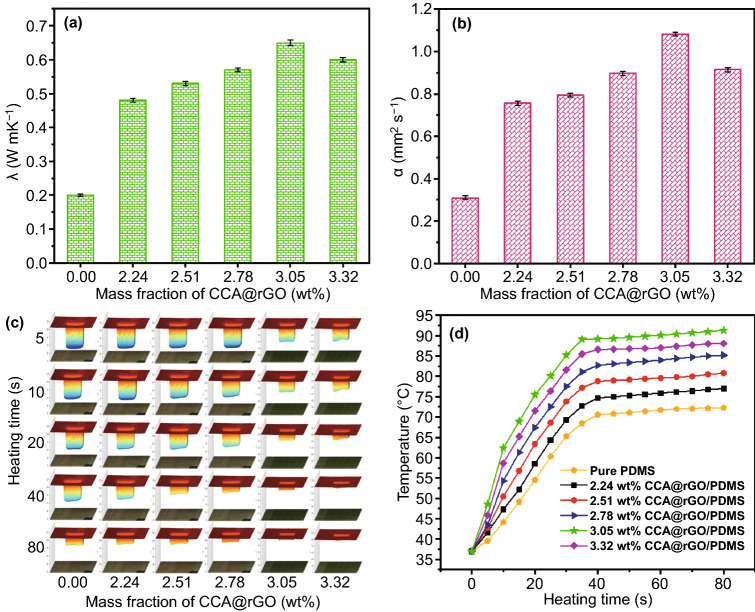
*λ* (**a**), *α* (**b**), 3D infrared thermal images (**c**) and surface temperature curves *vs* heating time (**d**) of the CCA@rGO/PDMS EMI shielding composites

Figure 6 shows the *λ* (a), thermal diffusivity (*α*, b), 3D infrared thermal images (c) and surface temperature curves *vs* heating time (d) of the CCA@rGO/PDMS EMI shielding composites. Figure 6a, b shows that *λ* and *α* of CCA@rGO/PDMS EMI shielding composites both tend to increase and then decrease as the amount of CCA@rGO increases. When the loading of CCA@rGO is 3.05 wt%, the CCA@rGO/PDMS EMI shielding composites have the largest *λ* (0.65 W mK^-1^) and *α* (1.082 mm^2^ s^-1^), which are 3.3 and 3.4 times higher than *λ* (0.20 W mK^-1^) and *α* (0.3185 mm^2^ s^-1^) of pure PDMS. This is because, as the loading of CCA@rGO increases, rGO gradually wraps the CCA fibers to form the 3D double-layer thermal conductivity network with the skin-core structure, which improves the thermal conductivities of CCA@rGO/PDMS EMI shielding composites. However, as the amount of CCA@rGO increases further, the internal rGO of CCA@rGO tends to agglomerate, which reduces the density of the thermal conductivity network inside the CCA@rGO/PDMS electromagnetic shielding composites [56, 57]. However, with the further increase in the loading of CCA@rGO, the rGO inside CCA@rGO tends to agglomerate, which decreases the density of the thermal conductivity network inside the CCA@rGO/PDMS EMI shielding composites, thus adversely affecting the *λ* and *α* of the CCA/PDMS EMI shielding composites [58].

As shown in Fig. 6c, the heat flow conduction rate is significantly higher inside the CCA@rGO/PDMS EMI shielding composites compared to the CCA/PDMS EMI shielding composites for the same temperature thermal stage and heating time, indicating their excellent thermal conductivities [59]. Meanwhile, with the increase in the amount of CCA@rGO, the heat flow conduction rate inside the CCA@rGO/PDMS EMI shielding composites becomes faster and then slower, indicating that the appropriate amount of CCA@rGO (3.05 wt%) is beneficial to further improving the thermal conductivities of the CCA@rGO/PDMS EMI shielding composites, which is consistent with the experimental results of Fig. 6a, b. In addition, the heat flow is uniformly conducted inside the CCA@rGO/PDMS EMI shielding composites, indicating the relatively uniform dispersion of CCA@rGO in the CCA@rGO/PDMS EMI shielding composites (consistent with Fig. 3d).

The surface temperature change of the CCA@rGO/PDMS EMI shielding composites is divided into two stages as the heating time increases (Fig. 6d). The first stage is the 0 to 40 s heating time period, where the surface temperature of the CCA@rGO/PDMS EMI shielding composite increases rapidly. This is mainly attributed to the low initial temperature of the CCA@rGO/PDMS EMI shielding composites, which causes the large temperature difference between them and the heat table, and therefore, the heat propagation rate is fast [60]. The second stage is the 40 to 80 s heating time period, where the surface temperature of the CCA@rGO/PDMS EMI shielding composites increase slowly. This is mainly attributed to the fact that after 40 s of heating, the temperature of the CCA@rGO/PDMS EMI shielding composites start to increase and the temperature difference between them and the hot table are smaller, so the heat propagation rate become slower [61]. It is also observed that the heating rate of the surface temperature of the CCA@rGO/PDMS EMI shielding composites tend to increase and then decrease in the first heating stage with the increase in the loading of CCA@rGO. In the case of both heating times is 40 s and the loading of CCA@rGO is 3.05 wt%, the surface temperature of CCA@rGO/PDMS EMI shielding composites reach the maximum value of 89.2 °C indicates that the appropriate CCA@rGO (3.05 wt%) is beneficial to efficiently enhance the thermal conductivities of CCA@rGO/PDMS EMI shielding composites [62].

### Mechanical Performances

**Fig. 7:**
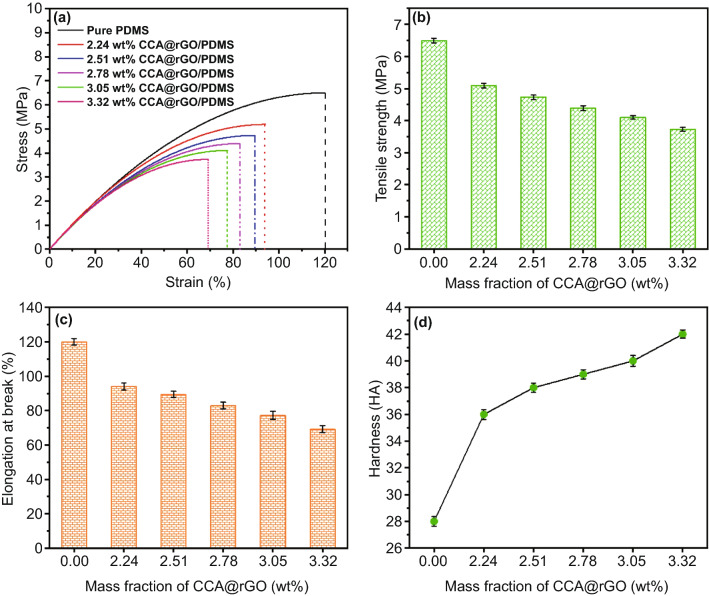
Stress–strain curves (**a**), tensile strength (**b**), elongation at break (**c**) and hardness (**d**) of the CCA@rGO/PDMS EMI shielding composites

Figure 7 shows the stress–strain curves (a), tensile strength (b), elongation at break (c) and hardness (d) of the CCA@rGO/PDMS EMI shielding composites. The tensile strength and elongation at break of the CCA@rGO/PDMS EMI shielding composites show a decreasing trend with the increase in the loading of CCA@rGO (Fig. 7a-c). When the loading of CCA@rGO is 3.05 wt%, the tensile strength and elongation at break of CCA@rGO/PDMS EMI shielding composites are 4.1 MPa and 77.3%, respectively, which are 36.9% and 35.6% lower than the tensile strength (6.5 MPa) and elongation at break (120%) of pure PDMS. It is mainly attributed to more two-phase interfaces (weak interfacial connections) between rGO and CCA, rGO and rGO, and CCA@rGO and PDMS matrix with the increase in CCA@rGO loading. It is easy to develop microcracks and voids inside the CCA@rGO/PDMS EMI shielding composites, resulting in the reduction of bond strength. When subjected to external forces, its internal defects will become stress concentration points and rapidly trigger the expansion and fracture of internal microcracks, thus reducing the tensile strength and elongation at break of CCA@rGO/PDMS EMI shielding composites.

As shown in Fig. 7d, the hardness of the CCA@rGO/PDMS EMI shielding composites illustrates a gradual increase with the increase in the loading of CCA@rGO. When the loading of CCA@rGO is 3.05 wt%, the hardness of CCA@rGO/PDMS EMI shielding composites reach 42 HA, which is 50% higher than that of pure PDMS (28 HA). This is mainly attributed to the fact that the network density of the rigid skeleton CCA@rGO gradually increases with the loading of CCA@rGO, forming more hard two-phase interfacial layers with the PDMS matrix, which effectively hinders the deformation of the CCA@rGO/PDMS EMI shielding composites under pressure, resulting in the increase in the hardness.

**Fig. 8:**
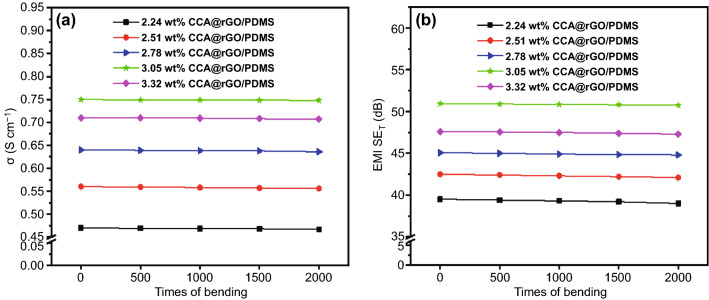
*σ* (**a**) and EMI SE_T_ (**b**) values of CCA@rGO/PDMS EMI shielding composites after bending fatigue

Figure 8 shows the *σ* (a) and EMI SE_T_ (b) results of CCA@rGO/PDMS EMI shielding composites after bending fatigue. The *σ* and EMI SE_T_ of CCA@rGO/PDMS EMI shielding composites show a slight decrease with the increase in the number of bending fatigues. When the bending fatigue reach 2000 times, the *σ* and EMI SE_T_ of CCA@rGO/PDMS EMI shielding composites are 0.745 S cm^−1^ and 50 dB (3.05 wt% CCA@rGO), respectively, which were only 0.7% and 2.0% lower than the *σ* (0.75 S cm^−1^) and EMI SE_T_ (51 dB) of the CCA@rGO/PDMS EMI shielding composites without bending fatigue, which indicates that the CCA@rGO/PDMS EMI shielding composites have good bending fatigue resistance.

### Thermal Stabilities

**Fig. 9:**
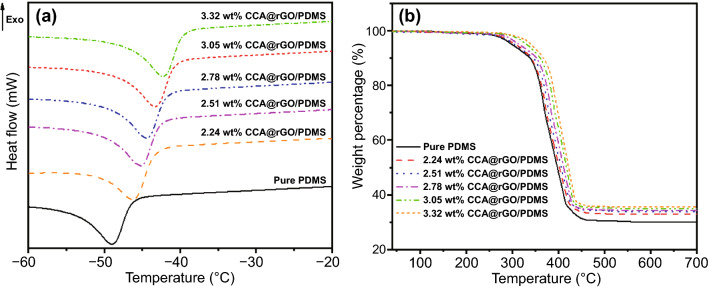
DSC curves (**a**) and TGA curves (**b**) of the CCA@rGO/PDMS EMI shielding composites

Figures 9a, b shows the DSC and TGA curves of the CCA@rGO/PDMS EMI shielding composites, respectively, and Table 1 shows the corresponding thermal characteristic data. Figure 9a and Table 1 show that the *T*_*g*_ of the CCA@rGO/PDMS EMI shielding composites gradually increases with the increase in the loading of CCA@rGO. When the loading of CCA@rGO is 3.05 wt%, the *T*_*g*_ of CCA@rGO/PDMS EMI shielding composites is −43.4 °C, which is 5.7 °C higher than that of pure PDMS. This is mainly attributed to the fact that the hard two-phase interfacial layer between CCA@rGO and PDMS matrix increases with the increase in CCA@rGO loading, which restricts the movement of PDMS molecular chains and makes *T*_*g*_ increase [63]. As shown in Fig. 9b and Table 1, the *T*_*5*_, *T*_*30*_ and the corresponding *T*_*HRI*_ of the CCA@rGO/PDMS EMI shielding composites show a gradual increase with the increase in the CCA@rGO loading. When the loading of CCA@rGO is 3.05 wt%, the *T*_*5*_, *T*_*30*_ and the corresponding *T*_*HRI*_ of the CCA@rGO/PDMS EMI shielding composites are 318.1, 394.4 and 178.3 °C, respectively. which are 25.8, 362.5 and 163.9 °C higher than The *T*_*5*_, *T*_*30*_ and *T*_*HRI*_ of the pure PDMS (292.3, 362.5 and 163.9 °C). It is mainly attributed to the fact that the introduction of rGO with excellent heat resistance can help improve the heat resistance of CCA@rGO/PDMS EMI shielding composites [64]. Meanwhile, the good interfacial compatibility between CCA@rGO and PDMS matrix can effectively prevent the oxygen penetration and thermal degradation behavior of CCA@rGO/PDMS EMI shielding composites [65, 66]. The synergy of the two aspects leads to the significant improvement in the heat resistance of CCA@rGO/PDMS EMI shielding composites compared to pure PDMS.

**Table 1:** Thermal characteristic data of the CCA@rGO/PDMS EMI shielding composites

Simples	*T*_*g*_ (^o^C)	Weight loss temperature (^o^C)	*T*_HRI_* (^o^C)	
		*T*_*5*_	*T*_*30*_	
PDMS	−49.1	292.3	362.5	163.9
2.24 wt% CCA@rGO/PDMS	−46.3	307.5	381.3	172.4
2.51 wt% CCA@rGO/PDMS	−45.4	310.2	384.6	173.9
2.78 wt% CCA@rGO/PDMS	−44.5	313.4	388.6	175.7
3.05 wt% CCA@rGO/PDMS	−43.4	318.1	394.4	178.3
3.32 wt% CCA@rGO/PDMS	−42.6	321.6	398.8	180.3

The sample’s heat resistance index is calculated by Eq. (1):

*T*_*HRI*_ = 0.49 [*T*_*5*_+0.6 (*T*_*30*_-*T*_*5*_)] (Eq. (1))

*T*_*5*_ and *T*_*30*_ are corresponding decomposition temperature of 5% and 30% weight loss, respectively

## Conclusions

rGO was successfully wrapped on the surface of CCA to form CCA@rGO with the 3D double-layer conductive network skin-core structure, and its 3D conductive network structure was not significantly damaged during backfilling with PDMS. When the loading of CCA@rGO is 3.05 wt%, CCA@rGO/PDMS EMI shielding composites have the best EMI SE_T_ (51.0 dB). At this time, the CCA@rGO/PDMS EMI shielding composites have outstanding thermal conductivities (*λ* is 0.65 W mK^−1^), excellent mechanical properties (tensile strength and hardness are 4.1 MPa and 42 HA, respectively) and excellent thermal stabilities (*T*_*HRI*_ of 178.3 °C). Excellent EMI shielding performances and thermal stabilities, as well as good thermal conductivities, make CCA@rGO/PDMS EMI shielding composites have great application prospects in lightweight, flexible electromagnetic shielding composites and portable and wearable electronic devices.

## Supplementary Information

Below is the link to the electronic supplementary material.

Supplementary file1 (PDF 361 KB)
