# Supplementary material for: Lightweight, Flexible Cellulose-Derived Carbon Aerogel@Reduced Graphene Oxide/PDMS Composites with Outstanding EMI Shielding Performances and Excellent Thermal Conductivities
Source: Nanomicro Lett. 2021 Mar 16;13:91. doi: 10.1007/s40820-021-00624-4 (PMC8006522; doi:10.1007/s40820-021-00624-4)
Supplement: Supplementary file 1 — Supplementary file1 (PDF 361 KB) [file 40820_2021_624_MOESM1_ESM.pdf]

Supporting Information for

Lightweight, Flexible Cellulose-Derived Carbon Aerogel@Reduced Graphene Oxide/PDMS Composites with Outstanding EMI Shielding Performances and Excellent Thermal Conductivities

Ping Song[1, #], Bei Liu[1, #], Chaobo Liang[1], Kunpeng Ruan[1], Hua Qiu[1], Zhonglei Ma[1], Yongqiang Guo[1], Junwei Gu[1, *]

[1]MOE Key Laboratory of Material Physics and Chemistry under Extraordinary Conditions, Shaanxi Key Laboratory of Macromolecular Science and Technology, School of Chemistry and Chemical Engineering, Northwestern Polytechnical University, Xi'an 710072, P. R. China

#Ping Song and Bei Liu contributed equally to this work

*Corresponding authors. E-mail: gjw@nwpu.edu.cn & nwpugjw@163.com (J. Gu)

S1 Experimental Section

S1.1 Main Materials

polydimethylsiloxane (PDMS) was provided by Momentive Performance Materials Inc. (Shanghai China). Desilted cotton was purchased from Yanggu Jingyanggang Sanitary Materials Factory (Shandong China). sodium hydroxide (NaOH) and urea (CH_4N_2O) were purchased from Guangdong GHTECH Co., Ltd. (Guangdong China). Graphite flake (325 mesh, 99.8%) was provided by Alfa Aesar Co. Ltd. (Shanghai China). Potassium persulfate ($K_2S_2O_8$, ≥99%), phosphorus pentoxide (P_2O_5, ≥98%), potassium permanganate ($KMnO_4$, ≥99%), sulfuric acid (H_2SO_4, 98%), hydrochloric acid (HCl, 37 wt%) and hydrogen peroxide (H_2O_2, 30 wt%) were all purchased from Beijing Chemical Factory (Beijing, China). All the chemicals were used as-received without any further treatment.

S1.2 Characterizations

Fourier Transform infrared (FTIR) spectroscopy of the samples was captured on a Bruker Tensor 27 equipment (Bruker Co., Germany) with thin films on KBr. X-ray photoelectron spectroscopy (XPS) of the samples was carried out using PHI5400 equipment (PE Co., UK). X-ray diffraction (XRD) of the samples was obtained on a Phillips PW3040-MPD diffractometer. Raman spectroscopy was performed using Alpha300R (WITec Co., Germany). Differential scanning calorimetry (DSC) was conducted using a DSC1 (Mettler-Toledo Co., Switzerland), under nitrogen atmosphere at a heating rate of 10 °C/min. Thermogravimetric analyses (TGA) of the samples were carried out under air atmosphere using STA 449F3 (NETZSCH C Co., Germany) over a temperature ranging from 40 to 1000 (10 °C/min). Morphologies of the samples were observed by scanning electron microscope (SEM, VEGA3-LMH, ESCAN Co., Czech Republic). A Fluke infrared thermal imager (Ti 300, Fluke) was used for the thermal measurement on a plate at a constant temperature of 90 °C. Direct current (DC) electrical conductivity of the samples was measured by a four-probe method at room temperature and the dimension of the samples was 22.86×10.16×3.00 mm^3. EMI shielding parameters of the samples were tested by VNA (MS4644A, Anritsu) using wave-guide method at X-band (8.2-12.4 Hz) according to ASTM D5568-08, and the corresponding dimension was 22.86×10.16 ×3.00 mm^3. A thermal

constant analyzer (Hot Disk TPS2200, AB Co., Sweden) was used to measure the thermal conductivities of the samples according to the standard of ISO 22007-2: 2008. The tensile strength and elongation at break were determined *via* electronic universal testing machine (CMT 6303, Shenzhen Xinsansi Testing Instrument Co., Ltd. China) according to the standard of GB/T 528-2009, and five dumbbell shaped specimens of each samples were prepared with the thickness of 2 mm at the stretching speed of 200 mm/min. Shore harnesses were valued through shore A hardness tester (ALX-AC, Shanghai Aolong Xingdi Testing Equipment Co., China) according to ASTM D2240-2004 standard.

S2 Characterizations on CA and CCA

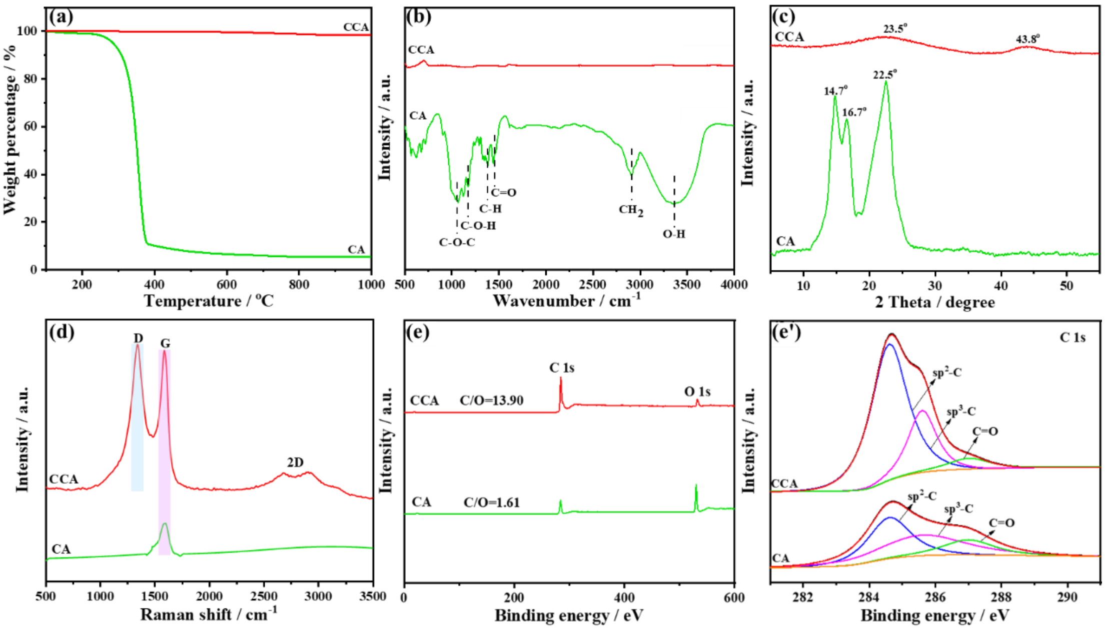

Fig. S1 TGA (**a**), FTIR (**b**), XRD (**c**), Raman (**d**), XPS spectra (**e**) and high-resolution C 1s (**e'**) of CA and CCA

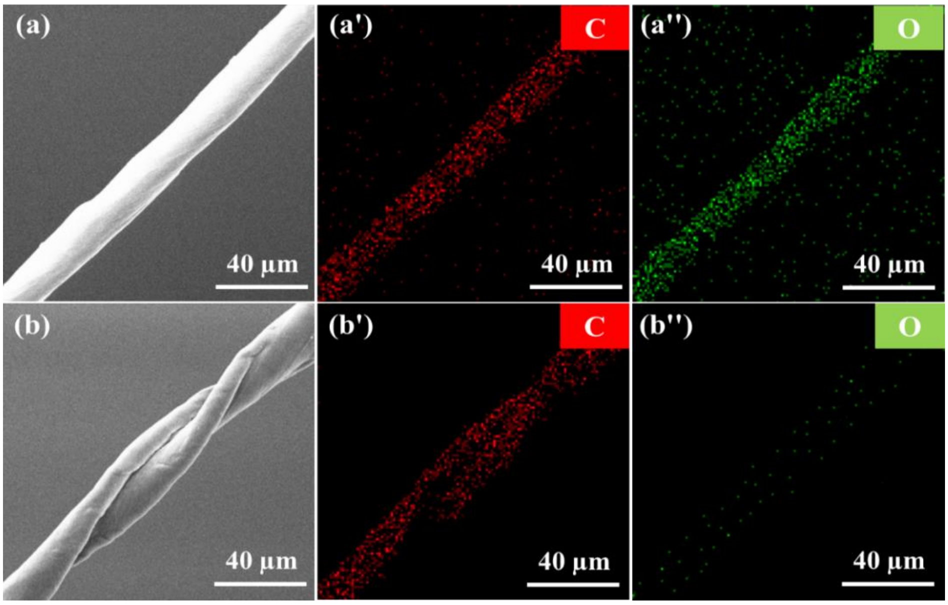

Fig. S2 SEM images of CA with elemental mapping images of C and O (**a-a"**) and CCA with elemental mapping images of C and O (**b-b"**)
